# Dual immunotherapy alternating with anti-PD-1 antibody plus liposomal doxorubicin show good efficacy in prostate epithelioid hemangioendothelioma: a case report

**DOI:** 10.3389/fimmu.2024.1384111

**Published:** 2024-06-14

**Authors:** Jie Zhang, Qin Ye, Xudan Yang, Tenglong Li, Shan Huang, Ping Zhou, Yumei Feng, Hao Liu, Ke Xie

**Affiliations:** ^1^ Department of Oncology, Sichuan Academy of Medical Sciences and Sichuan Provincial People’s Hospital, School of Medicine, University of Electronic Science and Technology of China, Chengdu, Sichuan, China; ^2^ Department of Oncology, Chengdu BOE Hospital, Chengdu, Sichuan, China; ^3^ Department of Pathology, Sichuan Academy of Medical Sciences and Sichuan Provincial People’s Hospital, School of Medicine, University of Electronic Science and Technology of China, Chengdu, Sichuan, China

**Keywords:** prostate epithelioid hemangioendothelioma, immunotherapy, PD-1 inhibitor, liposomal doxorubicin, nivolumab, ipilimumab

## Abstract

Epithelioid hemangioendothelioma is a rare vascular malignancy, and currently, there is no standard treatment regimen for this disease and existing treatment options have limited efficacy. In this case report, we present a patient with lung and lymph node metastases from prostate epithelioid hemangioendothelioma who achieved a significant partial response. This was accomplished through alternating nivolumab therapy with ipilimumab and liposomal doxorubicin, resulting in a progression-free-survival more than 6 months to date. The treatment was well-tolerated throughout. Our report suggests that dual immunotherapy alternating with anti-PD-1antibody plus doxorubicin may be a potential treatment modality for epithelioid hemangioendothelioma. However, larger sample studies are necessary to ascertain the effectiveness of this treatment strategy and it is essential to continue monitoring this patient to sustain progression-free survival and overall survival.

## Introduction

Epithelioid hemangioendothelioma (EHE) is a rare vascular malignancy that originates from vascular endothelial cells (1) and can manifest in various parts of the body, primarily in soft tissues (limbs), bones, liver, and lungs. It occurs only rarely in other parts of the body, such as the prostate (2) and pleura (3). It occurs in fewer than one in a million cases and constitutes less than 1 percent of all vascular tumors (4). For patients presenting with local, regional, and distant disease, 3-year survival rates were observed to be 79.7%, 70.7%, and 46.0%, respectively, with the majority of deaths occurring within the first two years (5). Prostatic epithelioid hemangioendothelioma is a rare pathological type of prostate malignancy, compared with the well-known prostate adenocarcinoma. According to the WHO classifications of urogenital and male genital tumors (5th edition), prostate malignancies are categorized into two main groups: epithelial tumors and mesenchymal tumors specific to the prostate, with adenocarcinoma remaining the most predominant disease (6), while rare pathological types also include EHE and lymphoma (7). Surgery remains the primary treatment for localized prostate malignancies, while for advanced tumors, the acceptable treatment options include chemotherapy, endocrine therapy, PARP inhibitors (8) and radiometabolic approaches (9, 10). A number of researchers have reported that immunotherapy did not show significant efficacy in prostate cancer (11). However, its efficacy against prostatic epithelioid hemangioendothelioma has not been explored, therefore, new effective therapeutic drugs and strategies need to be further explored.

In this paper, we present a rare case of prostate epithelioid hemangioendothelioma in which nivolumab treatment was alternated with ipilimumab and doxorubicin liposomes, resulting in a significant partial response. As of now, the patient has not experienced significant disease progression, but has maintained a persistent partial response (PR) and progression-free survival (PFS) for nearly seven months, and we are continuing to follow up with this patient.

## Case report

A 65-year-old man was admitted to the hospital with a history of “frequent urination and urgency persisting for six months, which worsened with distension and testicular pain over the last two months”. In January 2023, the patient began experiencing urinary tract irritation symptoms, including frequent urination and urgency, without apparent causes. By May 2023, these symptoms had escalated, accompanied by swelling, testicular distension, and pain. During a clinic visit in July 2023, a digital rectal examination was performed as part of the physical examination. The examination revealed a first-degree enlargement of the prostate with a smooth surface, unclear demarcation, hardness, palpable irregular nodules, and a shallow central sulcus. A contrast-enhanced CT scan of the entire abdomen revealed a soft tissue mass in the bladder rectal depression, measuring approximately 8cm x 5.8cm. The lesion showed unclear demarcation from the posterior bladder wall, prostate, seminal vesicles, left ureteral pelvic segment, left obturator internal muscle, and lower rectal segment, with associated upper ureteral dilation hydrops. Diagnostic imaging indicated a cystistic rectal fovea mass, raising consideration for neoplastic lesions.

**Figure 1:**
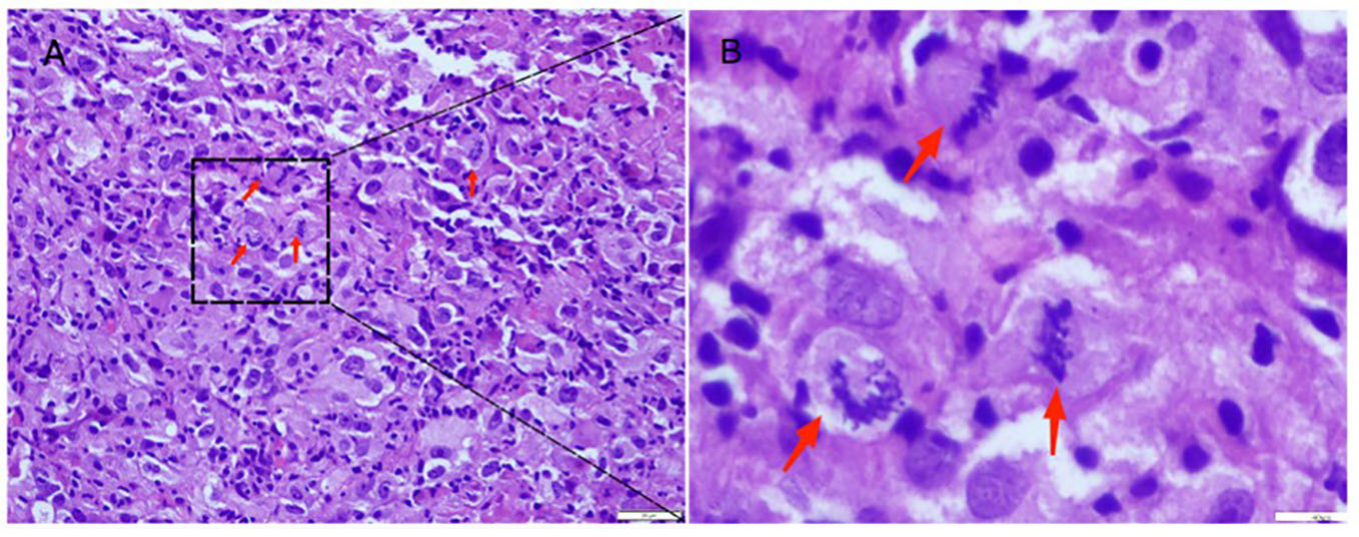
Hematoxylin-eosin staining of prostate tumors. **(A)** Red arrows indicate obvious mitotic signs signifying malignancy (200X). **(B)** The image is a magnified view at 400X, Red arrows indicate a significant nuclear atypia.

**Figure 2:**
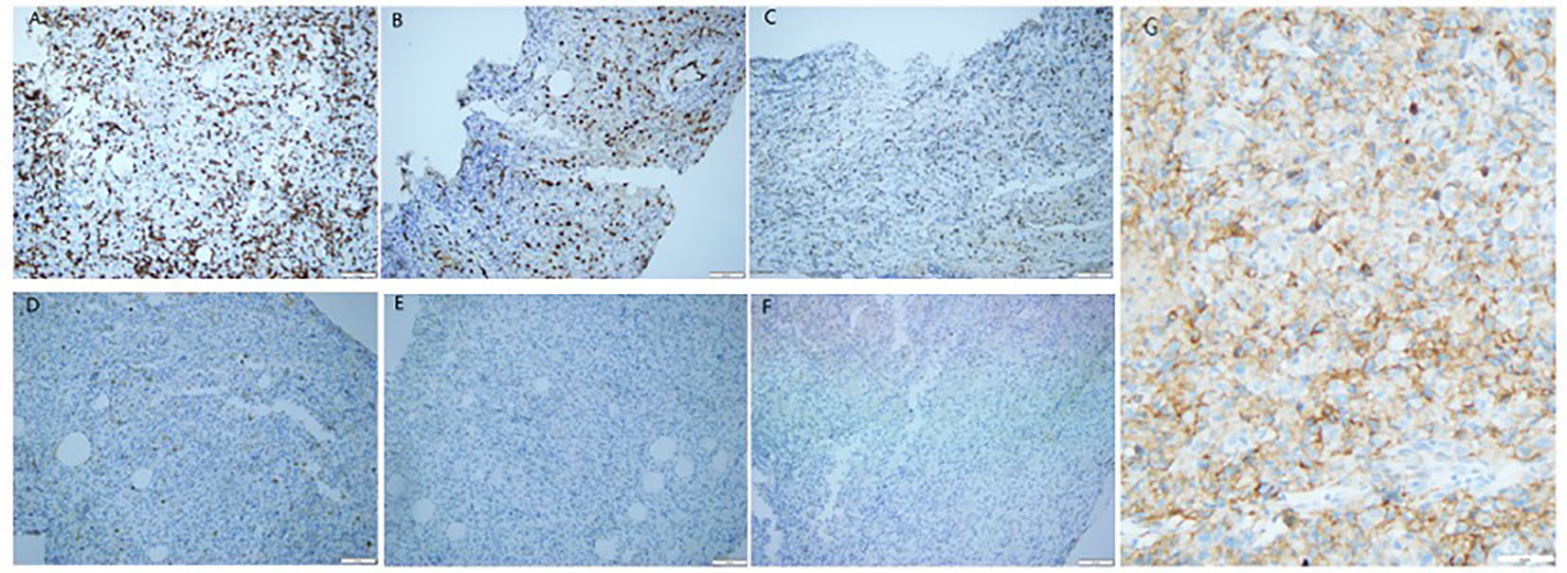
Immunohistochemical staining of the primary tumor revealed prostate epithelioid hemangioendothelioma. Tumor cells exhibited positivity for CD3 **(A)** (12), ERG **(B)** (13)and TIA1 **(C)** (14), while being negative for CK **(D)** (13), NKX3.1 **(E)** (15), and EMA **(F)** (16). PD-L1: CPS≥10 **(G)**. (**A-G** 200X; **A-C** markers denote association with vascular endothelial cells; the negativity of the **D** marker suggests a potential epithelial origin; the negative expression of the **E** marker excludes prostate adenocarcinoma; the **F** marker is rarely expressed in EHE.).

Prostate biopsy with immunohistochemistry revealed involvement in the left and right peripheral prostate with anterior, middle and posterior parts, left and right internal glandular areas, transition area, and apex. The puncture disclosed multiple nuclear atypic epithelioid/round cell infiltrates supporting malignancy (**Figure 1**), but the immunophenotype did not align with epithelial tumors and lymphomas. The immunohistochemistry (IHC) results were as follows: CD34-, CD68 background cells+, DES-, ERG+, HMB45-, MPO-, PAX5-, SALL4-, TIA1 background partial cells+, CD3 background T cells+, CD20-, CD30-, CDX2-, CK-, EMA-, GATA3-, NKX3.1-, P63-, S100-, Ki67 about 70%, PD-L1 (25%≤TC<50%,CPS≥10). The *in situ* hybridization (ISH) results showed EBER1/2 (**Figure 2**). Following a PET-CT systemic metabolic examination, a diagnosis of prostatic epithelioid hemangioendothelioma with lung and lymph node metastasis was established. Starting from 2023–08-02 and continuing on 2023–09-13 and 2023–11-03, the patient underwent nivolumab immunotherapy (360 mg) combined with ipilimumab (50 mg) for 1, 3, and 5 cycles. Additionally, a regimen of nivolumab 360 mg combined with liposomal doxorubicin (50 mg) was administered on 2023–08-26, 2023–10-07 and 2023–11-24 for 2, 4, and 6 cycles. The patient received intensity-modulated radiation therapy (IMRT) radiotherapy for a malignant prostate tumor from 2023–08-28 to 2023–10-06 at a PGTV radiation dose of 5040 cGy/28F (**Supplementary Figure 1**).

**Figure 3:**
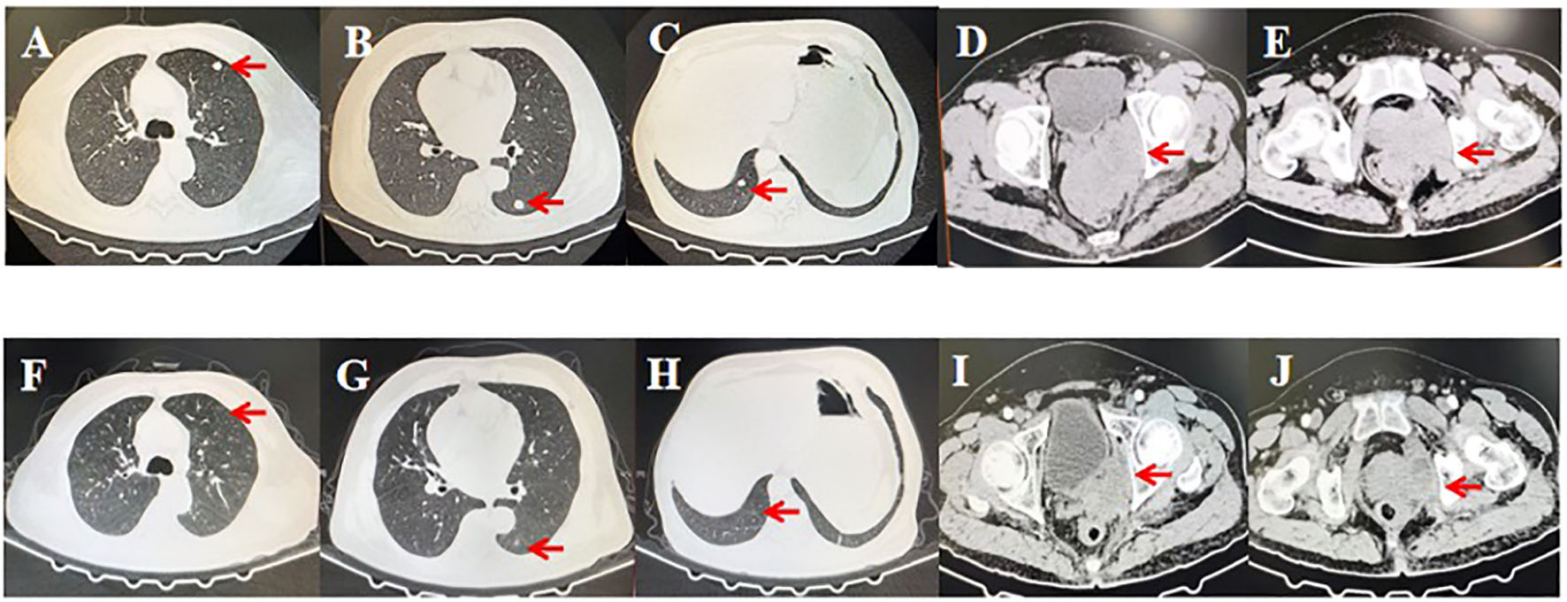
Computed tomography scans before and after treatment with nivolumab alternating with ipilimumab and doxorubicin liposomes. **(A-E)** Lung involvement and primary lesions of the prostate before initiating treatment. **(F-J)** Substantial reduction in lesions after completing six cycles of treatment. Red arrows indicate the lesions.

In November 2023, an efficacy assessment of the patient revealed a significant reduction in primary lesions and a notable decrease in lung metastases (**Figure 3**).

Throughout the treatment period, the patient underwent continuous monitoring for hematological markers and clinical signs, including blood parameters, liver and kidney function, heart function, immune-related indicators and skin reactions. To date, the patient has completed six cycles of treatment and shown a sustained response for more than seven months. Most importantly, only minor adverse events, such as grade I gastrointestinal reactions, were observed during the treatment, and no treatment-related adverse events, such as infusion reactions, cardiotoxicity, pulmonary toxicity, or hepatotoxicity occurred. Symptomatic treatment effectively improved the observed symptoms.

**Figure 4:**
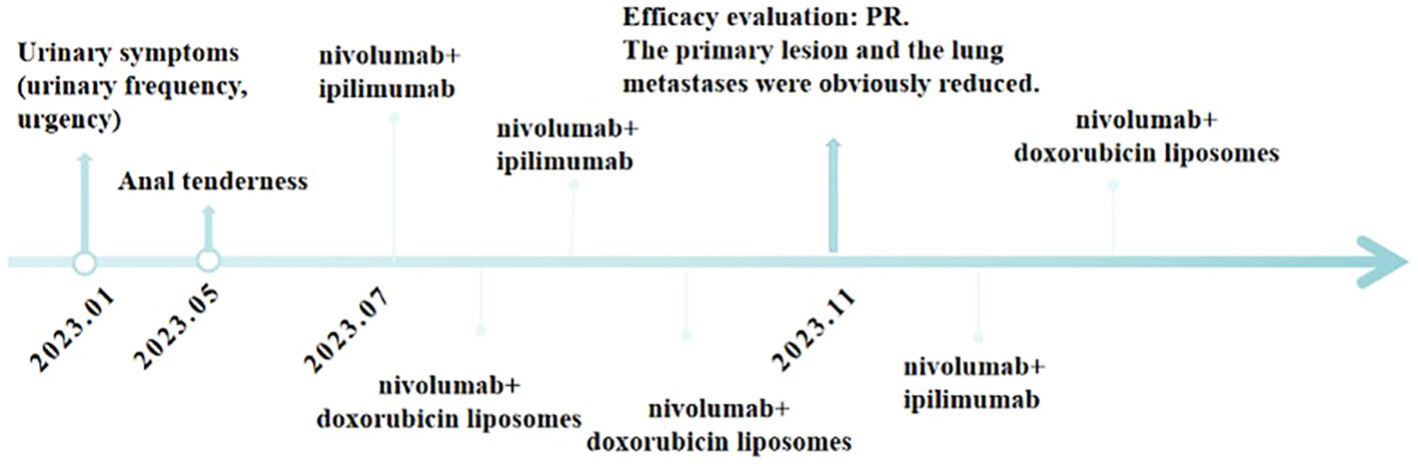
Timeline of major clinical events in the patient’s treatment since the onset of clinical symptoms.

It is worth mentioning that the patient’s serum creatinine levels were significantly elevated before treatment, suggesting postrenal renal insufficiency due to compression caused by the prostate tumor, as determined by nephrology consultation. Therefore, we do not consider renal dysfunction in this patient as a contraindication for anti-tumor treatment. Relevant indicators of renal function did not deteriorate further during the course of treatment, and after symptomatic treatment, the symptoms improved. The final conclusion, reached after discussions in the pharmacy department, was that the observed changes did not indicate drug toxicity. The course of treatment of the patient is shown in **Figure 4**.

## Discussion

As a rare tumor, there is currently no established standard for the optimal treatment of patients with prostatic epithelioid hemangioendothelioma. Treatment approaches for EHE vary, considering factors such as the tumor’s location, extent of involvement, presence of metastases, and individual patient characteristics. Currently, multimodal therapy (17), incorporating high-quality surgery, chemotherapy, radiotherapy, or a combination of these is the primary treatment strategy, although this is based on limited evidence and may differ depending on lesion sites or pathological features. Systemic treatment remains a recommendation for advanced epithelioid hemangioendothelioma, with certain drugs having been shown to have some efficacy. Vascular endothelial growth factor (VEGF) inhibitors such as sorafenib (18), pazopanib (19), and bevacizumab have been shown to be potentially effective in EHE treatment. One study of fifteen patients with epithelioid hemangioendothelioma treated with sorafenib (18) reported a median survival time of 124 days and a 9-month progression-free rate of 30.7%. Another study demonstrated that the administration of pazopanib (19)to a female patient with hepatic epithelioid hemangioendothelioma resulted in stable pulmonary nodules. Additionally, a minor reduction in tumor size was observed in the liver, accompanied by progressive calcification and tumor shrinkage. These findings suggest the potential involvement of VEGF in EHE growth. Reports also indicated that bevacizumab, when combined with capecitabine (20), and other drugs like thalidomide (21) and doxorubicin (22), have shown potential positive outcomes in hepatic EHE treatment. Sirolimus, a novel mTOR inhibitor, demonstrated benefit in the treatment of EHE, although it has not yet received official approved for sarcoma (23). Trametinib has also played a role in treating and improving symptoms in EHE patients (24). These studies demonstrated the potential efficacy of multiple treatment options for EHE, but the number of cases was small, particularly in hepatic EHE, and, consequently there is no universally accepted standard treatment regimen. Thus, the treatment approach for prostatic epithelioid hemangioendothelioma warrants further exploration.

Considering the notable impact of immunotherapy on various advanced solid tumors and the minimal side effects of the treatment, it deserves further study. The continue successes of immunotherapy have signaled a revolutionary shift in cancer treatment. It is thought that, the efficacy of this therapeutic approach is largely attributable to the antibody blockade of immune checkpoint regulators (25), focusing on the programmed cell death-1 (PD-1)/programmed cell death-Ligand 1 (PD-L1) and cytotoxic T-lymphocyte-associated protein 4 (CTLA-4) axis (26). This approach enhances the recognition and killing ability of immune cells, thus thwarting the immune escape of tumor cells and achieving anti-tumor effects (26). In the case of CTLA-4, the FDA-approved drug ipilimumab has demonstrated significant efficacy towards various cancers, including prostate, cervical, colon cancer, non-small cell, lung cancer, gastric, pancreatic, ovarian, urothelial carcinoma, and melanoma (27). The regulatory roles of PD-L1 and CTLA-4, along with their co-regulatory mechanisms, contribute to synergistic antitumor effects (28). In the Checkmate Study 227, the combination of nivolumab plus ipilimumab was recommended as an effective first-line treatment for patients with metastatic non-small cell lung cancer. The study reported a five-year overall survival (OS) rate of 24%, a progression-free survival (PFS) rate of 24.5 months, and a five-year OS rate of 39% during a follow-up of more than 61.3 months (29). The Checkmate Study 067 demonstrated that the combination of nivolumab and ipilimumab continued to enhance survival in patients with advanced melanoma in the first line. At a 7.5-year follow-up, nearly half of the patients were still alive, and the double-free group achieved a median OS of 72.1 months, marking the longest OS reported in a phase 3 clinical trial for advanced melanoma (30). It should be noted that some studies have reported that immunotherapy for prostate cancer was not significantly effective (31, 32). However, treatments involving pembrolizumab (33) and toripalimab (34) have demonstrated benefits in epithelioid hemangioendothelioma, making immunotherapy a viable alternative strategy for patients with widespread metastatic epithelioid hemangioendothelioma. The difference in treatment between the two may be due to the differing pathological types, as well as the low incidence of hemangioendothelioma, resulting in limited data on the immunotherapy research. The therapeutic efficacy of this case might also be correlated with the expression level of PD-L1.

Doxorubicin (DOX) is a potent anticancer agent commonly used in chemotherapy. It demonstrates a median progression-free survival of 4.2 months (95% CI: 3.7–4.8) and a median overall survival of 15.7 months (14.0–17.8) after six cycles (35). While playing a significant role in soft tissue sarcoma treatment, its dose-limiting nature due to cardiotoxicity has prompted the development of liposomal doxorubicin (36). Doxorubicin is encapsulated in polyethylene glycol-coated liposomes to form doxorubicin liposomes, which can evade surveillance by the mononuclear phagocyte system, prolong retention time in the bloodstream, and exploit the enhanced permeability and retention (EPR) effect of tumors to promote drug accumulation at tumor sites. Consequently, this encapsulation method reduces myocardial drug accumulation (37) and enhances the convenience of clinical treatment. Currently, liposomal doxorubicin is widely employed in the treatment of various conditions, including soft tissue malignant tumors, breast cancer (38), ovarian cancer (39), and others. As epithelioid hemangioendothelioma is believed to originate from the vascular endothelium with mesenchymal characteristics, the introduction of pegylated liposomal doxorubicin into EHE treatment (40) has shown positive responses, particularly in hepatic hemangioendothelioma cases (41), because it can reach higher concentrations in the liver and spleen (40). Therefore, given doxorubicin’s effectiveness against sarcomas of similar mesenchymal origin, it was chosen for our treatment approach. Given the current limited research on EHE treatment, systemic therapy or multimodal combination therapy is considered the primary choice for patients with advanced epithelioid hemangioendothelioma (17). Thus, because of this patient’s advanced tumor burden, we chose the regimen of doxorubicin in combination with other drugs for combating the tumors.

Alternate treatment regimens are now also being applied to a variety of diseases (42), harnessing the complementary effects of different drugs and specific pharmacological mechanisms. The objective is to maximize the killing of cancer cells, minimize toxicity, delay the onset of drug resistance, and prolong survival, especially for sarcomas, where various alternative treatment regimens have been employed. For instance, in Ewing’s sarcoma, alternating triple combination therapies such as vincristine, ifosfamide, and doxorubicin (VIA) and etoposide, ifosfamide, and cisplatin (VIP) have been tested (43). Regimens for soft tissue sarcomas have utilized alternating cycles of cyclophosphamide, doxorubicin, and vincristine (VDC) and ifosfamide and etoposide (IE) chemotherapy (44). Similarly, alternating regimens have been considered for EHE originating from the mesenchymal lineage. For nivolumab and ipilimumab combination therapy, ipilimumab was administered every six weeks, and nivolumab every three weeks. Consequently, we have designed a treatment regimen involving alternating immunotherapy drugs with chemotherapy and a second immunological agent.

Based on results of various studies, we adopted a treatment strategy involving the alternation of nivolumab with ipilimumab and liposomal doxorubicin, complemented by radiation therapy. Through our comprehensive approach, both the primary lesions in the prostate and the lung lesions exhibited significant reduction, leading to a substantial and sustained PR for the patient. Remarkably, the patient tolerated the entire treatment process well, experiencing no apparent adverse reactions or drug resistance. Consequently, the alternation of immunotherapy with liposomal doxorubicin and immunotherapy emerges as a potential treatment modality for epithelioid hemangioendothelioma. However, given the limited number of studies, it remains uncertain whether this approach can be applied on a larger scale. Further expansion of the sample size is essential to verify the efficacy of this method in treating epithelioid hemangioendothelioma. Additionally, in the future, we will continue to monitor the characteristic changes in this patient’s condition during treatment to obtain PFS and OS outcomes.

## Data availability statement

The original contributions presented in the study are included in the article/**Supplementary Material**. Further inquiries can be directed to the corresponding authors.

## Ethics statement

The studies involving humans were approved by Sichuan Academy of Medical Sciences and Sichuan Provincial People’s Hospital. The studies were conducted in accordance with the local legislation and institutional requirements. The participants provided their written informed consent to participate in this study. Written informed consent was obtained from the individual(s) for the publication of any potentially identifiable images or data included in this article.

## Author contributions

JZ: Conceptualization, Data curation, Writing – original draft. QY: Conceptualization, Data curation, Writing – review & editing. XY: Writing – original draft, Formal analysis. TL: Writing – original draft, Resources. SH: Writing – original draft, Resources. PZ: Writing – original draft, Resources. YF: Writing – original draft, Resources. HL: Investigation, Supervision, Validation, Funding acquisition, Writing – review & editing. KX: Investigation, Supervision, Validation, Funding acquisition, Writing – review & editing.
