# Supplementary material for: Dual immunotherapy alternating with anti-PD-1 antibody plus liposomal doxorubicin show good efficacy in prostate epithelioid hemangioendothelioma: a case report
Source: Front Immunol. 2024 Jun 14;15:1384111. doi: 10.3389/fimmu.2024.1384111 (PMC11211375; doi:10.3389/fimmu.2024.1384111)
Supplement: Supplementary file 1 [file DataSheet_1.pdf]

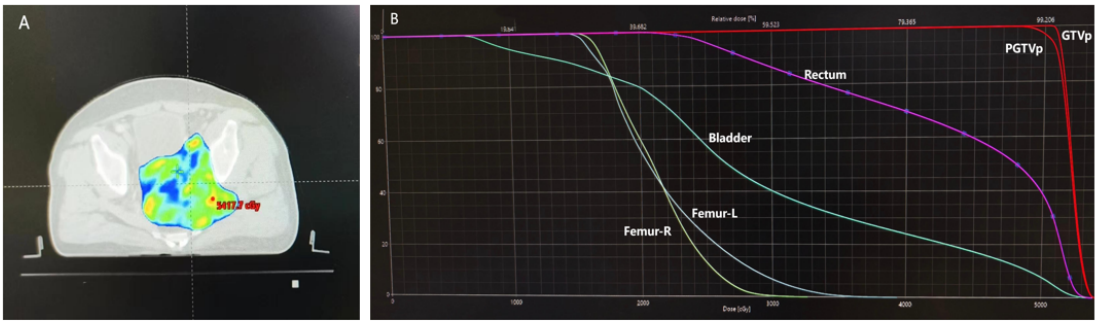

Supplementary figure 1 Administration of IMRT for the primary prostate lesion, with a prescribed dose of 5040 cGy/28F. (A) The blue line delineates the 5040 cGy area, while the red indicates the dose hot-spot. (B) The dose and volume histogram (normal tissue within specified limits).
